# Most Favored Nation Pricing and Affordability of GLP-1RAs for Obesity Treatment in Medicare

**DOI:** 10.1001/jamanetworkopen.2026.13098

**Published:** 2026-05-15

**Authors:** David D. Kim, Jennifer H. Hwang, A. Mark Fendrick, Dariush Mozaffarian

**Affiliations:** 1 Department of Medicine and Public Health Sciences, University of Chicago, Chicago, Illinois; 2 Section of General Internal Medicine, Department of Medicine, University of Chicago, Chicago, Illinois; 3 Department of Internal Medicine, University of Michigan, Ann Arbor; 4 Food is Medicine Institute and the Friedman School of Nutrition Science and Policy, Tufts University, Boston, Massachusetts

## Abstract

This economic evaluation examines the 10-year US pricing and affordability for glucagon-like peptide-1 receptor agonists (GLP-1RAs) for obesity treatment in Medicare.

## Introduction

In November 2025, the White House announced reductions in US prices for glucagon-like peptide-1 receptor agonists (GLP-1RAs) under a Most Favored Nation (MFN) policy, in exchange for expanded coverage of GLP-1RAs for obesity treatment. However, even at lower prices, the number of eligible individuals and the potential for long-term use in Medicare may result in a substantial fiscal impact. Building on our prior analyses,^1^ we estimated the potential 10-year Medicare budget impact of expanded access to GLP-1RAs for obesity at MFN prices under various scenarios.

## Methods

For this economic evaluation, we used a nationally representative, validated Diabetes, Obesity, Cardiovascular Disease microsimulation model.^2^ We estimated that 30 million Medicare Part D beneficiaries, including both current and those aging into eligibility, would meet clinical criteria for obesity treatment (body mass index [BMI; calculated as weight in kilograms divided by height in meters squared], ≥30; or ≥27 with 1 or more comorbidity).^1^ Individuals with diabetes or cardiovascular disease (CVD), who are already covered for GLP-1RAs in Medicare, were excluded from this analysis.

In the base case, we applied the announced Medicare price of $245 per month, assumed a 1-time treatment initiation (uptake) of 30% among the current and future cohorts, and modeled long-term adherence of 40% after the first year^3^ with gradual weight regain and loss of cardiometabolic benefits among individuals who discontinued treatment. With the anticipated loss of exclusivity for semaglutide in 2032 (year 7 of the analysis), we further modeled US-based, time-dependent additional discounts for drug price changes.^4^

Health care cost offsets were estimated by comparing lifetime health care spending among modeled individuals receiving GLP-1RAs plus lifestyle modification vs lifestyle modification alone, reflecting reductions in cardiometabolic risk, diabetes, CVD, and related complications. Medication costs were stratified into treatment initiation and maintenance phases.

Three-way sensitivity analyses varied monthly drug price ($150 for an oral pill at the lowest dose), $245 for Medicare, $350 for TrumpRx/self-pay), initial uptake (10% to 90%), and adherence (70% vs 40%). Higher adherence reflects the availability of more convenient oral formulations and lower patient cost-sharing. From a Medicare fiscal perspective, beneficiary out-of-pocket costs were not considered. This study followed the CHEERS reporting guidelines. The University of Chicago institutional review board determined that this study was exempt from review because it relied on the secondary analysis of publicly available data. Data were analyzed from November 2025 to February 2026 using R version 4.3.2 (R Foundation for Statistical Computing).

## Results

The analytic sample included 15.6 million current Medicare beneficiaries and 14.4 million individuals projected to become eligible over the next 10 years. The survey-weighted mean (SE) age was 64.5 (0.4) years, 54.1% were female, and the mean (SE) body mass index was 33.1 (0.3). In the base case ($245/mo, 30% uptake, 40% adherence), projected Medicare spending on GLP-1RAs for obesity treatment was $73.9 billion over 10 years ($23.4 billion initiation; $50.5 billion maintenance). Health care cost savings from clinical benefits were estimated at $56.3 billion, resulting in net increased spending of $17.6 billion. Estimated fiscal impacts were highly sensitive to treatment uptake: net spending varied from $5.0 billion at 10% uptake to $55.4 billion at 90% uptake (Figure). Price and adherence were key determinants of budget neutrality. At any uptake level, Medicare achieved net savings when monthly prices were lowered to $150, with long-term adherence remaining at 40% (Table).

**Figure.**
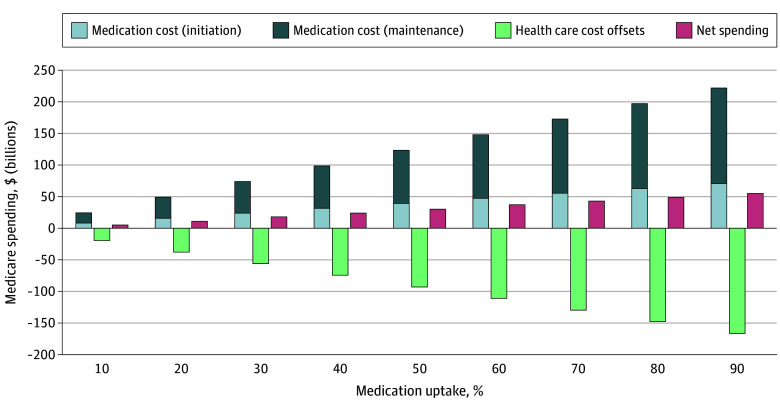
Ten-Year (2026-2035) Fiscal Impact of Medicare Coverage of Glucagon-Like Peptide-1 Receptor Agonists (GLP-1Ras) for Obesity Under the Most Favored Nation Price ($245/mo; 40% Long-Term Adherence) Stacked bars show projected 10-year Medicare spending across medication uptake scenarios (10% to 90%). Light blue denotes initiation-phase medication costs; dark blue denotes maintenance-phase medication costs. Green bars represent health care cost offsets from reductions in obesity-related morbidity. Red bars indicate net Medicare spending after accounting for cost offsets. Net spending increases with higher uptake, largely reflecting growth in maintenance-phase medication costs, while cost offsets rise proportionally with treatment adoption. Assuming loss of exclusivity for semaglutide in 2032 (year 7 of the analysis), US-specific, time-dependent additional discounts of 32%, 45%, 53%, and 59% were applied to the initial price of $245 per month in years 7 to 10.

**Table.** Ten-Year Estimated Fiscal Impact of Coverage of GLP-1RAs for Obesity Treatment: 3-Way Sensitivity Analysis

GLP-1RAs initial price, $/mo^a^^,^^b^	Medication cost, USD billions, $		Health care cost offsets, USD billions, $	Net cost, USD billions, $^d^
	Initiation	Maintenance		
10% Uptake				
40% Adherence				
350	11.2	24	−19.6	15.6
245	7.8	16.8	−19.6	5.0
150	4.8	10.3	−19.6	−4.5
70% Adherence				
350	11.2	42.1	−21.5	31.8
245	7.8	29.5	−21.5	15.8
150	4.8	18.1	−21.5	1.4
30% Uptake				
40% Adherence				
350	33.5	72.1	−56.3	49.3
245	23.4	50.5	−56.3	17.6^c^
150	14.4	30.9	−56.3	−11.0
70% Adherence				
350	33.5	126.4	−58.2	101.7
245	23.4	88.5	−58.2	53.7
150	14.4	54.2	−58.2	10.4
50% Uptake				
40% Adherence				
350	55.8	120.2	−93	83
245	39.1	84.2	−93	30.3
150	23.9	51.5	−93	−17.6
70% Adherence				
350	55.8	210.6	−94.9	171.5
245	39.1	147.4	−94.9	91.6
150	23.9	90.3	−94.9	19.3
70% Uptake				
40% Adherence				
350	78.1	168.3	−129.7	116.7
245	54.7	117.8	−129.7	42.8
150	33.5	72.1	−129.7	−24.1
70% Adherence				
350	78.1	294.9	−131.6	241.4
245	54.7	206.4	−131.6	129.5
150	33.5	126.4	−131.6	28.3
90% Uptake				
40% Adherence				
350	100.5	216.4	−166.4	150.5
245	70.3	151.5	−166.4	55.4
150	43.1	92.8	−166.4	−30.5
70% Adherence				
350	100.5	379.1	−168.3	311.3
245	70.3	265.4	−168.3	167.4
150	43.1	162.5	−168.3	37.3

Abbreviations: GLP-1RA, glucagon-like peptide-1 receptor agonist.

^a^ In 3-way sensitivity analyses, 3 monthly GLP-1RA price scenarios were evaluated: (1) $150 for the oral formulation (initial dose); (2) $245 reflecting Medicare pricing; and (3) $350 reflecting TrumpRx/self-pay pricing.

^b^ Assuming loss of exclusivity for semaglutide in 2032 (year 7 of the analysis), US-specific, time-dependent additional discounts of 32%, 45%, 53%, and 59% were applied to the initial price in years 7 to 10, respectively, across all price scenarios.

^c^ Base-case analysis assuming 30% uptake, 40% adherence, and a GLP-1RA price of $245 per month.

^d^ These estimates represent mean model outcomes under specified assumptions and should be interpreted as scenario-based projections rather than precise forecasts. Because our primary objective was to conduct deterministic sensitivity analyses of key policy-relevant parameters (eg, uptake, price, and adherence), we varied these inputs individually to illustrate their direct effects on total spending and net cost.

## Discussion

Our estimates suggest that the reduced MFN price of GLP-1RAs and accompanying coverage for obesity treatment in Medicare will likely increase drug spending, with only partial offsets from downstream health care savings. Although our model did not project achieving net savings at announced MFN prices of $245 per month over 10 years, cost neutrality could be achievable at substantially lower prices (eg, $150/mo). Higher long-term adherence increases cumulative net spending because weight loss benefits plateau while medication costs persist. Most GLP-1RAs expenditures also accrue beyond the initiation year as maintenance therapy. These projections suggest that alternative strategies to sustain weight loss and cardiometabolic benefits may warrant evaluation, including dose or frequency reduction, transition to lower-cost pharmacotherapies, and structured behavioral or nutrition programs.^5^ However, pragmatic trials are needed to determine the effectiveness of alternative weight maintenance strategies.

Policy uncertainty remains substantial. The legal authority to implement MFN pricing and its interaction with the Inflation Reduction Act’s Medicare Maximum Fair Price (MFP) framework remain unresolved. MFN prices, anticipated in 2026, may supersede the scheduled MFP for semaglutide ($274/mo) in 2027, but litigation or administrative revisions are possible. Moreover, the durability of the MFN policy depends on manufacturer responses, global pricing dynamics, and opaque international rebates.

A major limitation is that our estimates exclude costs associated with additional clinical visits and laboratory monitoring often needed during GLP-1RAs initiation and follow-up,^6^ which would further increase net spending and lower the drug price at which cost neutrality might be achieved. More evidence is needed on the effects of GLP-1RAs on health care utilization, particularly among obesity-only populations using second-generation GLP-1RAs.

## Notes

Supplement.
Data Sharing Statement

## References

[1] Hwang JH, Laiteerapong N, Huang ES, Mozaffarian D, Fendrick AM, Kim DD. Fiscal impact of expanded Medicare coverage for GLP-1 receptor agonists to treat obesity. JAMA Health Forum. 2025;6(4):e250905. doi:10.1001/jamahealthforum.2025.0905 40279111 PMC12032556

[2] Kim DD, Wang L, Lauren BN, . Development and validation of the US diabetes, obesity, cardiovascular disease microsimulation (DOC-M) model: health disparity and economic impact model. Med Decis Making. 2023;43(7-8):930-948. doi:10.1177/0272989X231196916 37842820 PMC10625721

[3] Rodriguez PJ, Zhang V, Gratzl S, . Discontinuation and reinitiation of dual-labeled GLP-1 receptor agonists among US adults with overweight or obesity. JAMA Netw Open. 2025;8(1):e2457349. doi:10.1001/jamanetworkopen.2024.57349 39888616 PMC11786232

[4] Serra-Burriel M, Martin-Bassols N, Perényi G, Vokinger KN. Drug prices after patent expirations in high-income countries and implications for cost-effectiveness analyses. JAMA Health Forum. 2024;5(8):e242530. doi:10.1001/jamahealthforum.2024.2530 39150730 PMC11329876

[5] Mozaffarian D, Agarwal M, Aggarwal M, . Nutritional priorities to support GLP-1 therapy for obesity: a joint Advisory from the American College of Lifestyle Medicine, the American Society for Nutrition, the Obesity Medicine Association, and The Obesity Society. Obesity (Silver Spring). 2025;33(8):1475-1503. doi:10.1002/oby.24336 40445127 PMC12304835

[6] Wing C, Cai S-T, Sacks DW, Simon KI. Do GLP-1 medications pay for themselves? National Bureau of Economic Research. Accessed April 10, 2026. https://ideas.repec.org/p/nbr/nberwo/34678.html

