# Supplementary material for: Most Favored Nation Pricing and Affordability of GLP-1RAs for Obesity Treatment in Medicare
Source: JAMA Netw Open. 2026 May 15;9(5):e2613098. doi: 10.1001/jamanetworkopen.2026.13098 (PMC13179543; doi:10.1001/jamanetworkopen.2026.13098)
Supplement: Supplement. — Data Sharing Statement [file jamanetwopen-e2613098-s001.pdf]

Data Sharing Statement

Data
Data available: No

Additional Information
Explanation for why data not available: We utilized publicly available information.
